# Aldehyde Dehydrogenases Expression in Corneal Epithelial Cells with Limbal Stem Cell Deficiency

**DOI:** 10.3390/ijms23074032

**Published:** 2022-04-05

**Authors:** Fawzia Bardag-Gorce, Alissa Diaz, Robert Niihara, Jeremy Stark, Daileen Cortez, Alexander Lee, Richard Hoft, Yutaka Niihara

**Affiliations:** 1 Department of Medicine, The Lundquist Institute, Torrance, CA 90502, USA; alissakdiaz@gmail.com (A.D.); robert.niihara@lundquist.org (R.N.); jeremy.stark@lundquist.org (J.S.); daileen.corteze@lundquist.org (D.C.); alexander.lee@lundquist.org (A.L.); rhoft@dhs.lacounty.gov (R.H.); yniihara@emmauslifesciences.com (Y.N.); 2 Emmaus Life Sciences Inc., Torrance, CA 90503, USA

**Keywords:** ALDH1A3, ALDH1A1, retinoic acid, CAOMECS, corneal epithelium reconstruction

## Abstract

Purpose: The purpose of the present study is to investigate the expression of aldehyde dehydrogenases (ALDHs) in rabbit corneas with limbal stem cell deficiency (LSCD) and corneas treated with cultured autologous oral mucosa epithelial cell sheet CAOMECS designed to reconstruct the ocular surface with LSCD. Methods: New Zealand white rabbit autologous oral mucosal epithelial cells were isolated from a buccal biopsy and cultured to be grafted back onto corneas of rabbit model of LSCD. Immunofluorescent staining and Western blot analysis were used to compare the expression of ALDH1A1 and ALDH1A3 in healthy, LSCD-diseased, CAOMECS treated corneas. Human oral mucosal and corneal epithelial cells (OMECS and CECs) were cultured and treated with retinoic acid (RA) to further investigate the expression of ALDHs. Results: In healthy corneas, ALDH1A1 and ALDH1A3 were markedly expressed in basal cells of corneal epithelium. In LSCD diseased corneas, ALDH1A1 and ALDH1A3 were markedly expressed in the conjunctivalized apical epithelial cells, the goblet cells, and the stroma. CAOMECS grafted corneas showed a decreased expression of ALDHs as compared to LSCD diseased corneas. Western blot analysis confirmed the up regulation of ALDH1A1 and ALDH1A3 expression in LSCD-diseased corneal epithelial cells. CAOMECS expressed low levels of ALDH1A1 and ALDH1A3, as compared to diseased CECs (D-CEC). When ALDH1A3 was up regulated by retinoic acid treatment in OMECS, Pax-6 expression was down regulated, suggesting a decrease in regenerative capacity when ALDH enzymes are up regulated. Conclusions: These findings report for the first time the up regulation of ALDH1A1 and ALDH1A3 in rabbit corneas with LSCD and document that CAOMECS grafting used to reconstruct corneal epithelium may reduce the expression levels of ALDH enzymes.

## 1. Introduction 

Our lab is currently working on developing cultured autologous oral mucosal epithelial cell sheets (CAOMECS) treatment for the ocular surface of patients with limbal stem cell deficiency. To treat bilateral limbal stem cell deficiency (LSCD) without the risk of rejecting an allogenic graft or causing damage to the donor fellow eye through partial biopsy of healthy limbal tissue, cultured autologous oral mucosal epithelial cell sheet (CAOMECS) is an excellent therapeutic choice, especially for bi-lateral LSCD. The biopsied buccal tissue contains stem cells that are cultured to produce a multilayered cell sheet that can be harvested intact and grafted back onto patient cornea without a carrier, avoiding the issues that come with carrier grafts [1]. Because these cell sheets can be grafted directly onto corneal surface and do not require sutures, there is less risk of stromal scarring and greater improvement of visual acuity for the patients [2]. Transplanted CAOMECS do not require sutures because it was cultured on a temperature-responsive cell culture-ware (CellSeed Inc. Tokyo Japan). When the temperature is lowered, the polymer of the culture-ware surface release CAOMECS with an intact extra cellular matrix, which results in fast adherence of CAOMECS to corneal surface. In pre-clinical study few sutures were used to prevent rabbits from damaging CAOMECS graft [3]. In clinical trial CAOMECS was secured with contact lenses [4].

Aldehyde dehydrogenases (ALDH) are NAD(P)^+^-dependent enzymes that catalyze the oxidation of a multitude of aldehydes to carboxylic acids [5], detoxifying and attenuating the oxidative stress aldehydes cause [6]. These enzymes are found throughout the body and they can have different functions, depending on their location. Aldehydes can be long-lived, highly reactive, and strongly electrophilic compounds that can form adducts with proteins, RNA, and DNA, which can result in the inactivation of enzymes, disruption in homeostatic regulation, DNA damage and cellular death [7]. Some aldehydes can play roles in normal physiological mechanisms, but the majority are cytotoxic and carcinogenic, especially those that are formed as a product of lipid peroxidation. 

Certain ALDH enzymes are highly expressed in the cornea in humans and other mammals, and upregulation of these proteins is a response to oxidative and electrophilic stress [8]. Although the function of ALDH in the cornea is not fully understood, it has been shown that these proteins protect the cornea from ultraviolet radiation and damage caused by reactive oxygen species [9]. ALDH enzymes scavenge the cornea for toxic aldehydes produced by lipid peroxidation [10]. The main ALDH enzymes that are highly expressed in human corneas are ALDH3A1 and those of the ALDH1 family (ALDH1A1, ALDH1A2, ALDH1A3) and account for about 3% of the soluble proteins in the cornea. Rabbit corneas mainly express ALDH1 enzymes (ALDH1A1, ALDH1A2, and ALDH1A3) but do not express ALDH3A1. It has been proposed that ALDH1 enzymes are primarily expressed in the corneal epithelium of species that lack ALDH3A1 to make up for its protective and antioxidant functions [8]. ALDH3A1 and ALDH1 enzymes have been known to be cytoplasmic proteins but have been shown more recently to be found in the nucleus as well, pointing to a possible role in the regulation of cell proliferation [9]. This finding indicates that ALDH enzymes not only protect the eye from oxidative stress, but, when located in the nucleus, that they may have a cell cycle regulatory function [9], pointing toward a possible relationship between their location in the nucleus and their function in cellular proliferation [11].

ALDH1A3 is also involved in corneal homeostasis [12] and plays an important role in the metabolism of vitamin A (retinal) and synthesis of retinoic acid (RA), a key regulator for corneal wound healing. The embryonic organogenesis role of RA in self-renewal, cellular proliferation and differentiation during ocular development is well-documented [13,14]. However, the role played by the ALDH1 family in adult tissue maintenance is much less understood.

In the present study, we investigated the expression of ALDH1A1 and ALDH1A3 in rabbit corneas with limbal stem cell deficiency and corneas treated with CAOMECS. 

## 2. Results

Cultured autologous oral mucosal epithelial cell sheets (CAOMECS) technology is an autologous cell sheet used to re-epithelize the ocular surface with LSCD [3,4,15,16]. In order to understand the mechanism and the efficacy of the designed treatment method, we needed to explore the way it affects corneal response to injury. The present study was designed to understand the expression of ALDH enzymes in corneas with LSCD and to explore the effects of CAOMECS grafting on the expression of ALDH enzymes in treated cornea.

A biopsy of rabbit buccal tissue was performed to isolate oral mucosal epithelial cells (OMECS). OMECS were cultured 2–4 weeks to produce CAOMECS as reported previously (3). CAOMECS was then grafted onto the rabbit ocular surface. Rabbits were followed for 6 months to examine the effectiveness of CAOMECS in regenerating corneal epithelium and improving the ocular surface, compared to LSCD corneas (sham) that did not receive CAOMECS.

The ALDH1 family of enzymes, namely ALDH1A1 and ALDH1A3, also labelled as retinaldehyde dehydrogenase 1 and 3, played an important role in oxidizing vitamin A (retinal) to retinoic acid (RA) that is required for corneal re-epithelialization and wound healing. They were the most expressed in the ocular surface. We explored the expression of ALDH1A1 and 1A3 in healthy, sham, and CAOMECS grafted rabbit corneas. 

### 2.1. Analysis of ALDH1A1 and 1A3 Expression:

Paraffin embedded tissue sections of rabbit corneas were used in immunofluorescent staining experiments to analyze ALDH proteins’ expression.

**Figure 1:**
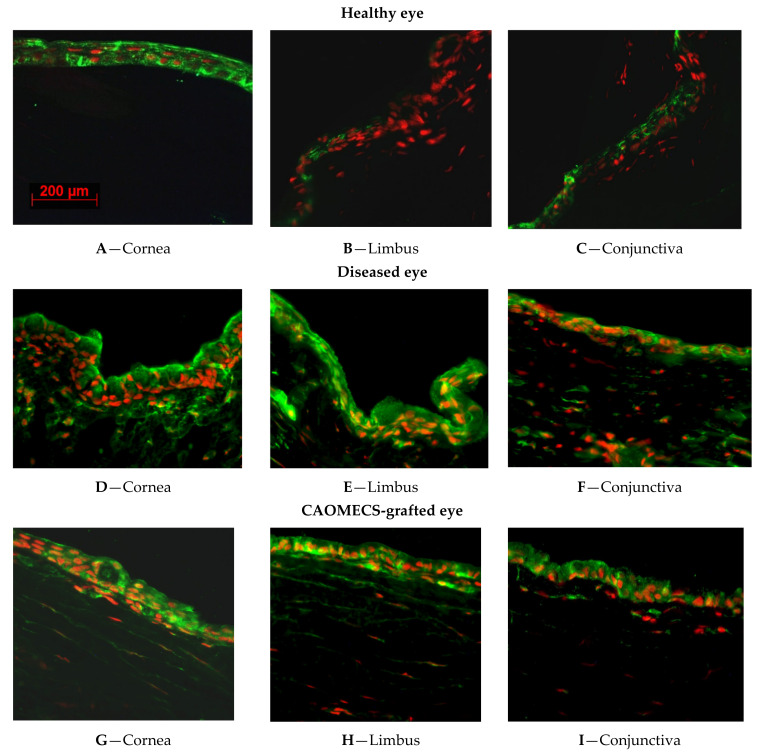
Immunofluorescent staining illustrated the expression pattern of ALDH1A1 in healthy cornea (**A**–**C**) in LSCD diseased (**D**–**F**; 9 months after LSCD induction) and CAOMECES grafted (**G**–**I**) New Zealand white rabbit corneas. ALDH1A1 expression is detected in green, and nuclei are detected in red. The included images are representation of a large sample size. Magnification is 40×.

Figure 1 shows the expression of ALDH1A1.

#### 2.1.1. ALDH1A1 Expression in Healthy Rabbit Corneal Tissues

The immunofluorescent staining revealed that ALDH1A1 was greatly expressed in the cytoplasm of corneal epithelial cells of healthy rabbit (Figure 1A). There was no positive staining in the limbus (Figure 1B), and less expression of ALDH1A1 over the conjunctiva (Figure 1C).

#### 2.1.2. ALDH1A1 Expression in LSCD Diseased Corneal Tissues

As we are exploring the different expression patterns of ALDH1A1 in corneal epithelial cells, it is important to understand the expression changes during the LSCD development. ALDH1A1 expression was present all over the cornea, limbus, conjunctiva and markedly increased in the stroma of the sham eye (Figure 1D–F) harvested 9 months after LSCD was induced.

#### 2.1.3. ALDH1A1 Expression in CAOMECS Grated Corneal Tissues

To reconstruct the ocular surface, CAOMECS was produced and grafted back onto rabbit corneas as previously described [3]. CAOMECS grafting reconstructs corneal epithelium and conferred protection from conjunctival cells invasion, and neovascularization [3]. As corneal epithelium was reconstructed with CAOMECS, there was less goblet cells and lower expression of ALDH1A1 (Figure 1G–I) than that seen in the LSCD diseased corneas (Figure 1D–F). In addition, ALDH1A1 expression was also reduced in the stroma, after CAOMECS grafting over the cornea, compared to the non-grafted cornea (shame eye) (Figure 1G–I).

**Figure 2:**
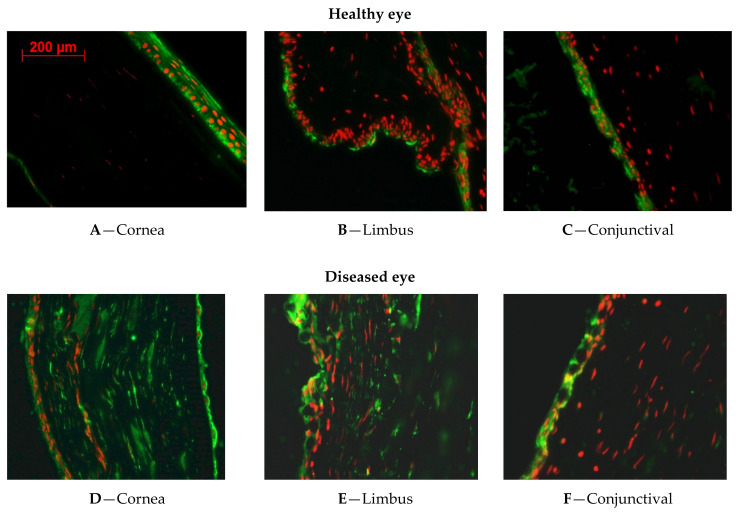

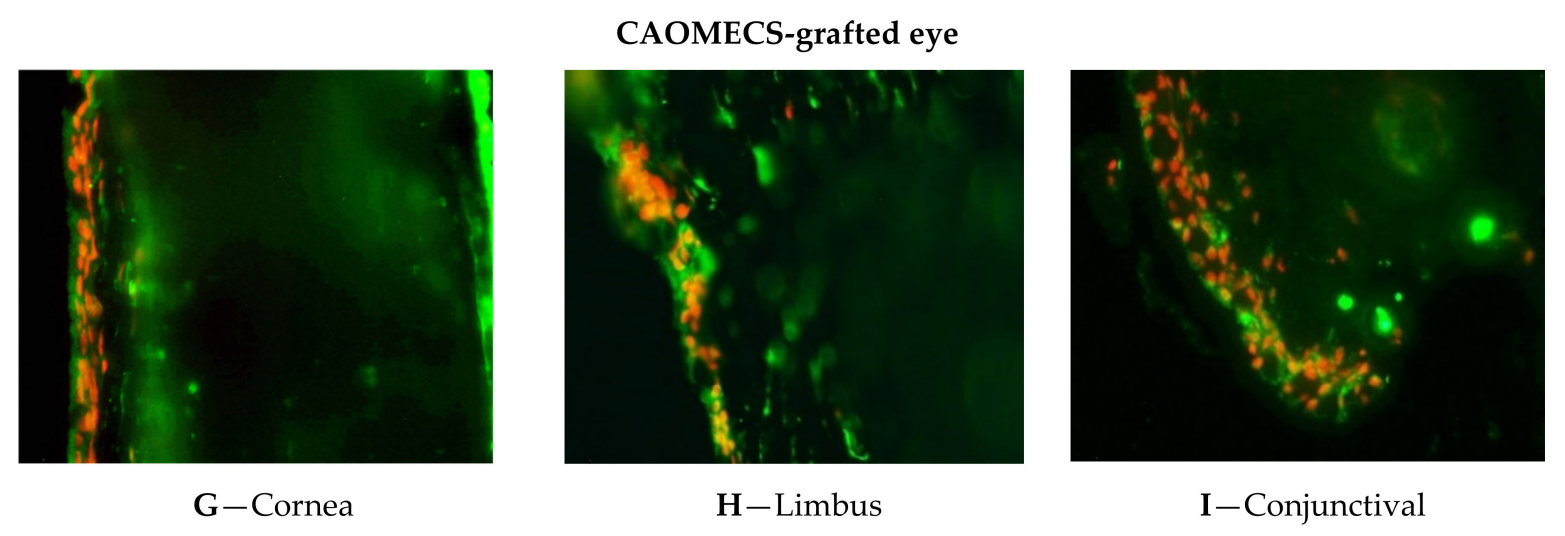
Immunofluorescent staining showing the expression pattern of ALDH1A3 in healthy (**A**–**C**), LSCD (**D**–**F**; 9 months after LSCD induction) and CAOMECES-grafted (**G**–**I**) New Zealand white rabbit corneas. ALDH1A1 expression is shown in green, and nuclei are shown in red. The included images are representations of many samples. Magnification is 40×.

Figure 2 shows the expression of ALDH1A3.

#### 2.1.4. ALDH1A3 Expression in Healthy Rabbit Corneal Tissues

In corneal epithelium, the results showed a marked expression of ALDH1A3 (Figure 2A). The limbus (Figure 2B) weakly expressed ALDH1A3 in the apical squamous cells, while the undifferentiated basal cells of the limbus did not express ALDH1A3. In the conjunctiva, ALDH1A3 was expressed in the cytoplasm and nuclei of conjunctival epithelial cells, as well as inside the goblet cells (Figure 2C).

#### 2.1.5. ALDH1A3 Expression in LSCD Diseased Corneal Tissues

Figure 2D shows that ALDH1A3 is expressed over the cornea, but also in the stromal. These results indicate that limbectomy changed the expression of ALDH1A3, mainly in the stroma. Figure 2E shows the area where the limbus underwent limbectomy. Conjunctival epithelial cells mixed with goblet cells markedly expressing ALDH1A3 invaded this area. The conjunctival epithelial cells were also positive for ALDH1A3 and goblet cells were numerous, bigger, and markedly expressing ALDH1A3.

#### 2.1.6. ALDH1A3 Expression in CAOMECS Grated Corneal Tissues

ALDH1A3 expression was still stable over the cornea (Figure 2G–I). Conjunctival epithelial cells showed a lower signal of ALDH1A3 (Figure 2I), compared to the sham eye (Figure 2F). By re-epithelializing corneal surface, CAOMECS grafting slightly decreased the expression of ALDH1A3 in the ocular surface. However, the expression of ALDH enzymes was still high in the stroma and endothelium (Figure 2D).

### 2.2. Semi-Quantitative Analysis of ALDH1A1 and ALDH1A3 Expression

**Figure 3:**
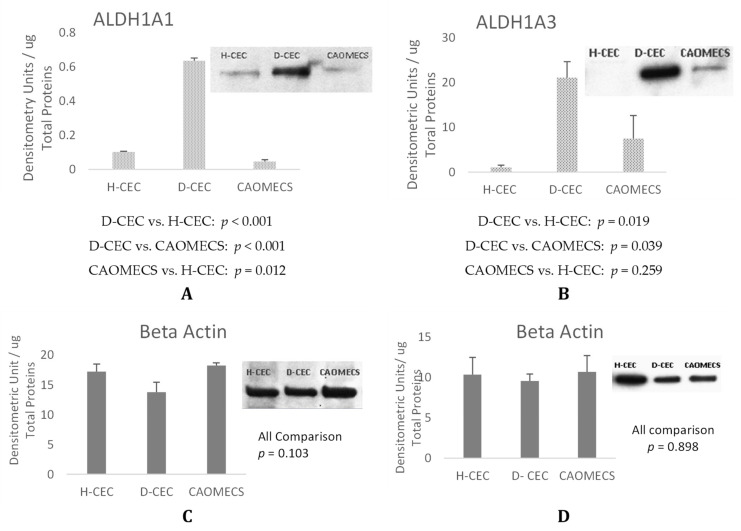
Expression levels of ALDH1A1 (**A**) and ALDH1A3 (**B**) in healthy CEC (H-CEC), diseased CEC (D-CEC) and in CAOMECS. Note that ALDH1A1 and ALDH1A3 were significantly increased in D-CEC as compared to H-CEC and CAOMECS. (**C**, **D)** are beta-actin levels measurement used as the loading control for the semi quantitative measurements used in this experiment. Values are shown as Mean ± SEM, *n* = 3. All Pairwise Multiple Comparison Procedures were done using Student-Newman-Keuls Method.

To further analyze ALDH1A1 and ALDH1A3 levels of expression, Western blot analysis was conducted. Healthy and LSCD diseased corneal epithelial cells were sampled from rabbit ocular surfaces and were compared to CAOMECS. Figure 3A shows that both ALDH proteins were up regulated in LSCD-diseased corneal epithelial cells (D-CEC) as compared to healthy corneal epithelial cells (H-CEC) and to CAOMECS. ALDH1A1 was significantly and highly expressed in D-CEC (Figure 3A). There was no significant difference between H-CEC and CAOMECS. ALDH1A3 levels of expression was up regulated in D-CEC (Figure 3B) without a significant difference. Similar to ALDH1A1, there was no significant difference between H-CEC and CAOMECS for ALDH1A3. It is possible that ALDH1A3 expression varied in D-CEC as there was an erosion of corneal epithelium caused by LSCD.

### 2.3. Retinoic Acid Induced the Expression of ALDH and Vice Versa

**Figure 4:**
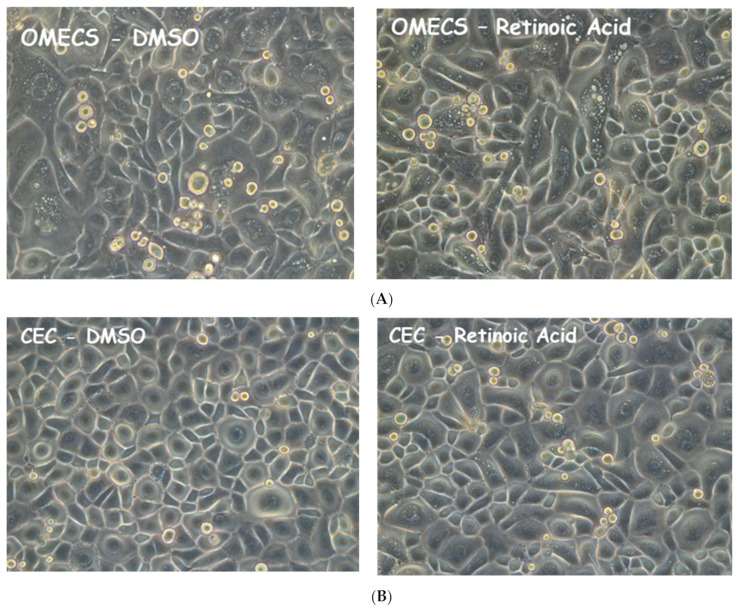
Cell culture of human oral mucosal and corneal epithelial cells (**A**,**B** respectively, OMECS and CEC). Cells were expanded and then treated with retinoic acid when 100% confluent. RA treatment was done for 24 h. Cells were then harvest and processed for biochemical analysis. The included images are representation of a large sample size. Magnification is 20×.

ALDH enzymes are required for the synthesis of retinoic acid (RA) that is essential for diverse biological and a potent regulator of cellular proliferation, differentiation, and apoptosis during embryogenesis and ocular development. ALDH1 enzymes are required for retinoic acid synthesis. Therefore, to understand the effect of ALDH induction after corneal injury, human CEC and OMECS were cultured and treated with 1 µM retinoic acid to up regulate the expression of these enzymes [17]. Figure 4 shows live cultured OMECS (A) and CEC (B), 24 h after treatment with RA. There was no difference in the morphology of the RA treated cells as compared to non-treated cells (DMSO).

**Figure 5:**
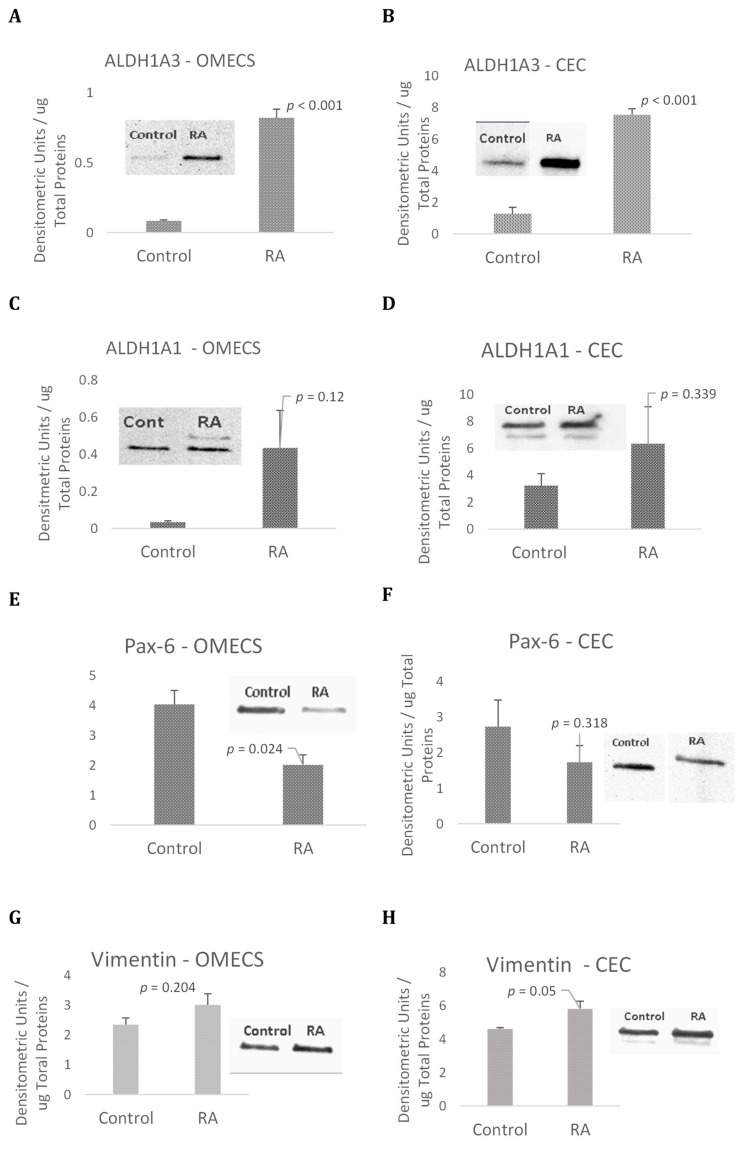

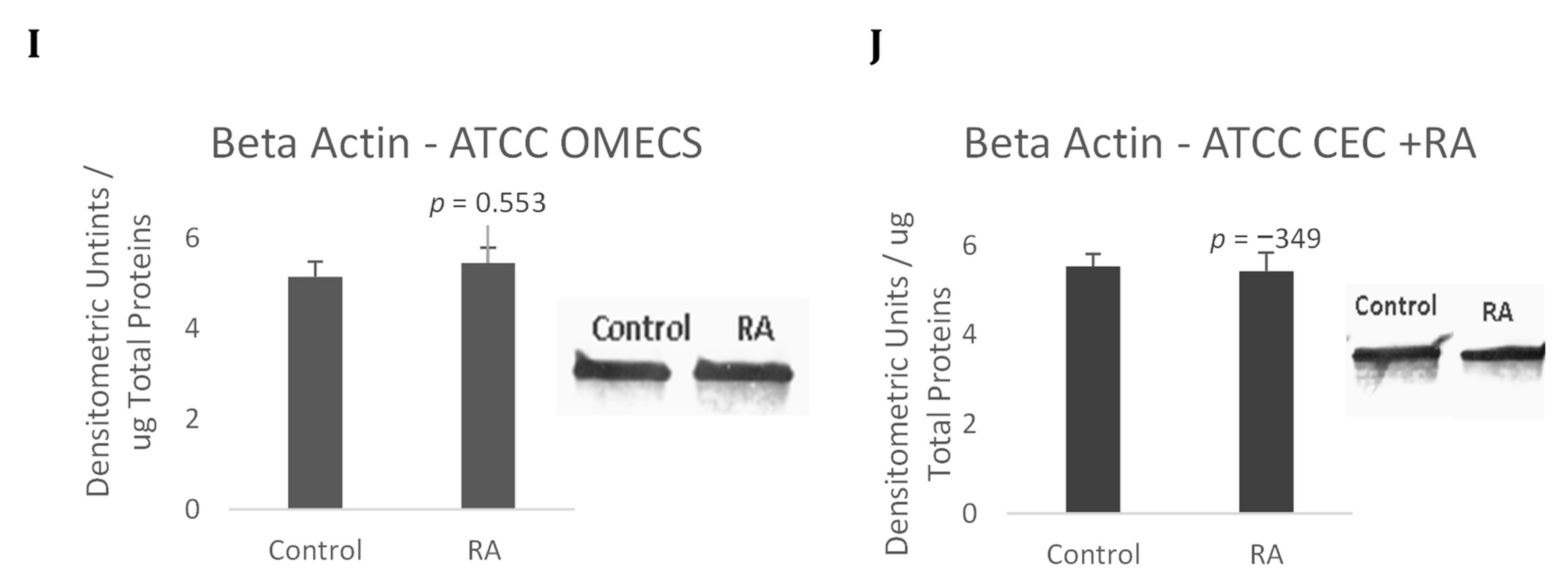
Cultured human oral mucosal and corneal epithelial cells (respectively, hOMECS and hCEC) were treated with retinoic acid (RA) for 24 hours. Cells were harvested and analyzed for the expression levels of ALDH1A3 (**A**,**B**), ALDH1A1 (**C**,**D**), Pax-6 (**E**,**F**), Vimentin (**G**,**H**) and Beta actin for protein loading controls (**I**,**J**). Values are shown as Mean ± SEM, *n* = 3. All Pairwise Multiple Comparison Procedures were done using Student-Newman-Keuls Method.

When retinoic acid was added to cell culture media, there was an increase in the expression of ALDH1 enzymes. This positive feedback loop has been documented to be happening at the transcriptional level yielding an increase of ALDH1 enzymes synthesis, especially ALDH1A3 [18]. Figure 5A,B show that ALDH1A3 was significantly up regulated in both OMECS and CEC cells treated with RA. ALDH1A1 expression was also up regulated in both cells (Figure 5C,D) with no significant difference (Figure 5C,D).

Experiments were then conducted to investigate the effects of RA treatment on the expression levels of Pax-6, a transcription factor that plays a major role in regulating cell cycle and cell fate of limbal and corneal epithelial stem cells. RA treatment decreased Pax-6 expression only in the OMECS (Figure 5E). Our results indicate that RA treatment increases ALDH1A3 expression, decreased Pax-6 levels in OMECS (Figure 5E). Pax-6 expression tends to decrease in CEC but the difference was not significant (Figure 5F).

It is possible that the number of progenitor cells positive for Pax-6 decreased, which promote a profibrotic phenotype reflected by the increase in the expression of Vimentin (Figure 5G,H).

**Figure 6:**
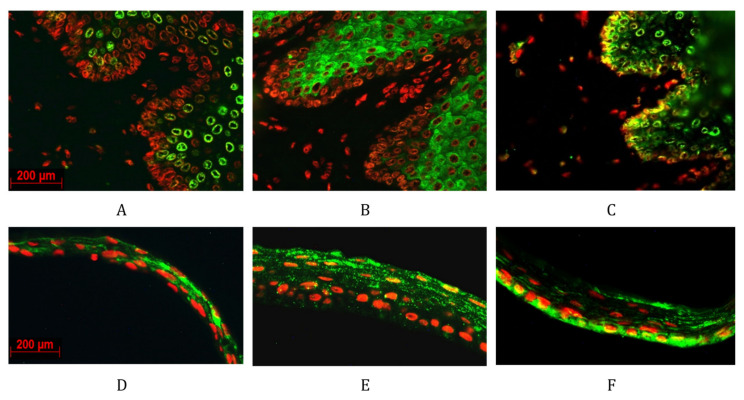
Immunofluorescent staining showed the expression pattern of ALDH1A1, ALDH1A3 and Pax-6 in New Zealand white rabbit oral mucosal (respectively **A**–**C**) and in rabbit CAOMECS (respectively **D**–**F**). ALDH enzymes and Pax-6 expression is detected in green, and nuclei are detected in red. The included images are representation of a large sample size. Magnification is 40×. The scal barr is 200um for all the pictures.

Figure 6 showed that both ALDHs did not stain the basal layer of oral mucosal tissue section. In contrast, Pax-6 stained mainly the basal layer of oral mucosa indicating that healthy progenitor stem cells are negative for ALDH1A1 and 1A3. Similarly, CAOMECS basal cells stained positive for Pax-6 (Figure 6F) and negative for ALDH1A1 and 1A3 (Figure 6D,E).

## 3. Discussion

The objective of this study was to explore the expression of ALDH enzymes in rabbit corneas with LSCD and to examine the effects of CAOMECS treatment in ALDH expression. Using the rabbit model of LSCD, we measured the expression of ALDH1A1 and ALDH1A3 in corneas of healthy, LSCD-diseased and CAOMECS grafted rabbit eyes.

In the healthy corneas, we noticed that ALDH1A1 and ALDH1A3 expressions were found in the apical squamous cells as well as in the basal and suprabasal cells. There was no expression of ALDH enzymes in the limbus and some expression in the apical cells of the conjunctiva. This result indicated that limbal stem cells are not positive for ALDH enzymes. ALDH1 enzymes were found on the apical side of the limbus, confirming previous publication showing that ALDH1 is in fact decreasing the proliferation of the cells. Indeed, the knockout of ALDH1 results in an increase of deltaNp63 expression in the limbus [10].

In the LSCD-diseased corneas or the sham corneas that did not receive CAOMECS, both ALDH1A1 and ALDH1A3 were highly expressed in the surface of the corneas. As LSCD causes corneal epithelium defects and erosion, ALDH enzymes expression was dominantly found in the invasive conjunctival epithelial and goblet cells. While the origin of ALDH overexpression is not fully known, conjunctival epithelial cells could be the source as these cells, along with goblet cells, markedly expressed both ALDH enzymes. However, as the stroma and endothelium also exhibited a high expression of ALDHs, it is not yet clear how exactly ALDHs are induced after corneal injury.

In CAOMECS grafted corneas, ALDH expression was reduced as compared to in LSCD-diseased corneas, but ALDH expression was still up regulated as compared to the control. Western blot analysis showed that the ALDH expression in CAOMECS was similar to that in healthy corneal epithelial cells, which was significantly lower than that in the LSCD-diseased. These results suggested that CAOMECS grafting seeded cells that show a low expression of ALDHs. Since CAOMECS grafting regenerated the ocular surface and decreased neovascularization as well as fibrosis [3], it is possible that CAOMECS grafting decreased the expression of both ALDH1A enzymes in corneal surfaces.

Several other studies have established that ALDH1A enzymes are expressed in the cytoplasm of epithelial cells. Stagos et al., [7] found that ALDH3A1 and ALDH1A1 were also localized in the nucleus of these epithelial cells. Different publication about ALDH1 enzymes showed that these enzymes are involved in eliminating the aldehyde formation in the cells, but also in maintaining the transparency of the cornea [7].

In LSCD, the corneal epithelium is being invaded by conjunctival epithelial cells, which divide and migrate to invade the central cornea, thus causing corneal haziness and eventually corneal blindness. Previously, we reported that there was an increase in expression of proangiogenic factors in the LSCD-diseased corneal epithelium [14]. This upregulation of ALDH1A1 and proangiogenic factors in the apical cells could be due to hypoxia or the inflammation that occurs after the limbectomy. High levels of HIF-1α have been shown to induce the expression of ALDH3 in corneal epithelial cells [19]. Recently, it was shown that endogenous retinoic acid generated in the mouse retina activates Sox9 in retinal pigment epithelia, which stimulates secretion of vascular endothelial growth factor (VEGF) and encourages blood vessel growth in the choroid [20].

The results from the semi-quantitative analysis confirmed those of the immunofluorescent analysis. ALDHs enzymes were up regulated in the LSCD-diseased corneal epithelial cells (month 3) as compared to healthy both rabbit CEC and rabbit CAOMECS. This is the first time reporting that ALDH enzymes are up regulated in corneas with LSCD animal model. Therefore, to understand ALDH up regulation in the injured ocular surface, human CEC and OMECS were cultured and treated with retinoic acid (RA). RA is a metabolite of Vitamin A (retinol) that is an important signaling molecule in regulating diverse developmental processes during ocular development. The ALDH enzymes are the enzymes responsible for RA synthesis [18] and their product that is RA [8] induces them. Increased levels of RA could contribute to the increased proliferation of epithelial cells including conjunctival epithelial cells invading the cornea in LSCD. Because RA is important to maintaining corneal homeostasis and regeneration, it may be increased during injury, which explains the increased expression of ALDH1A1. As expected, our results showed that exogenous RA treatment markedly and significantly up regulated the expression of both ALDH enzymes in both type of cells, hOMECS and hCEC.

The increase in the ALDH enzymes also indicated an up regulation of cell growth and differentiation, which possibly reflects a decrease in the number of stem cells in RA treated cells [21]. Pax-6 was decreased when ALDHs enzymes were increased only for OMECS cells, which supported our hypothesis that cell differentiation may be activated when ALDHs enzymes expression is high. Moreover, Vimentin staining showed that cells expressing high levels of ALDH1A1 and ALDH1A3 display a profibrotic phenotype, which was also found in rabbit corneal cells with LSCD. The fact that the basal cell of healthy limbus did not stain positive for ALDHs indicates that these ALDH enzymes are not a marker for normal and healthy limbal stem cells [22], but ALDH1 are expressed in more differentiated cells and in maintaining the cornea transparency [7,10].

After corneal injury, re-epithelialization, neovascularization and fibrosis all manifest simultaneously with other complicated pathophysiological process required for wound healing. Because the levels of ALDH1A1 expressed in the injured cornea exceeded those necessary for metabolism, it has been proposed that these enzymes may contribute to corneal tissues recovery in different way such as serving as cancer stem cell markers [23,24,25] contributing to epithelial cell hyper proliferation. This observation is supported by the fact that in our model of LSCD, PCNA and Ki67, biomarkers of proliferation, were found markedly expressed in the all layers of rabbit cornea with LSCD as previously reported [3]. In addition, alpha smooth muscle actin was found highly expressed in the LSCD diseased corneas and was reduced in the corneas that received CAOMECS [3]. CAOMECS grafted corneas were followed for 6 months before sacrificed and corneal tissue harvest. It is possible that ALDH expression in CAOMECS grafted corneas needed more time to be at the level of the healthy corneas.

## 4. Material and Methods

### 4.1. Animal Experiment

New Zealand white rabbits weighing 2.5–3 kg were used. They were maintained according to the Guidelines of Animal Care, as described by the National Academy of Sciences published by the Institute of Laboratory Animal Resources Commission on Life Sciences National Research Council. The experimental protocol was approved by the IACUC and performed as previously reported [3]. Rabbits were briefly sedated, subjected to lamellar limbectomy and followed for 3 months. The ophthalmologist evaluated the ocular surface and noted that LSCD was stable at 3 months. LSCD diseased corneas were then prepared for CAOMECS grafting by removing LSCD diseased corneal epithelial cells including pannus tissue, blood vessels and conjunctival cells. These harvest cells (D-CEC) were used for Western blot experiments. The LSCD diseased corneas were then shared in two groups, CAOMECS grafted corneas group and its control sham corneas group that did not receive CAOMECS graft. Six months later, corneas were harvested, fixed and examined with immunostaining. Therefore, Western blot experiments were conducted on LSCD diseased corneas at 3 months and immunohistochemistry experiments were conducted on corneas grafted with CAOMECS and the LSCD diseased sham cornea at 9 months.

### 4.2. Ethical Statement

Rabbit were housed and maintained under the supervision of the institutional veterinarian according to the Guidelines of IACUC (Institutional Animal Care and Use Committee). The research protocol was reviewed and approved by the IACUC. The project identification code is 20381-01 approved from 8/18/2011 to 8/31/2012.

### 4.3. Oral Mucosal Epithelial Cells Isolation and CAOMECS Engineering

Once LSCD was confirmed stable by the ophthalmologist, the rabbit’s oral mucosa was biopsied, and the specimen was incubated with Dispase II (Roche Diagnostics GmbH, Mannheim, Germany), to dissociate the tissues (as described in [14]). The epithelium was then peeled off from the lamina propria and subjected to trypsin digestion to dissociate the epithelium into isolated epithelial cells. The isolated epithelial cells were seeded on UpCell, a temperature-responsive culture ware (CellSeed Inc., Tokyo, Japan), in co-culture with Mitomycin C (MMC)-treated NIH/3T3 feeder cells [3,4,5,6,7,8,9,10,11,12,13,14]. After 2–4 weeks of cell culture, cell sheets grew as a multilayered epithelial tissue like. One cell sheet was grafted back onto the rabbit cornea and the other cell sheets were used for biochemical and histological analysis. CAOMECS grafted rabbits were then followed for 6 months to examine the therapeutic effects of the CAOMECS grafting [14].

### 4.4. Sampling of Corneal Epithelial Cells (CEC)

Rabbit corneal epithelial cells (CECs) were collected as previously reported [14]. Briefly, healthy (control, the day that LSCD was induced)) and diseased (LSCD, 3 months after the LSCD was induced) rabbit corneal epithelial cells were collected by exposing the corneas to 20% isopropyl alcohol. The corneas were then washed 3 times with saline and epithelial cells were scraped and removed from the corneal surface using a scalpel blade. The collected cells were then suspended in lysis buffer containing 20 mM Tris-HCl pH = 7.5, glycerol 10%, EGTA 1 mM, DTT 1 mM, protease and phosphatases inhibitor cocktail, as instructed by the supplier (Sigma, St Louis, MO, USA). Cell membranes were disrupted by pipetting 25 times the cells trough a hypodermic 18G needle.

### 4.5. Immunohistochemistry

Paraffin-embedded tissue sections (9 months after the LSCD was induced) were used to conduct immunohistochemical analysis. Tissue sections were stained using ALDH1A1 (R&D Systems, Inc. Minneapolis, MN, USA), ALDH1A3 (GeneTex, Inc. Irvine, CA, USA) and Pax-6 (Millipore Sigma—Life Science, St Louis, MI, USA) antibodies. Alexa Fluor® 488 donkey anti-mouse fluorophore conjugated secondary antibodies was used. Cell nuclei staining was performed using propidium iodide (Invitrogen, Eugene, OR, USA). Slides were analyzed using a Nikon 400 fluorescent microscope. Picture processing and analysis was performed using Adobe Photoshop CS5 (Adobe, New York, NY, USA).

### 4.6. Human Primary Oral Mucosal and Corneal Epithelial Cells

Commercially available human primary oral mucosal and corneal epithelial cells were cultured as instructed by the supplier (ATCC, Manassas, VA, USA) and treated with 1 µM retinoic acid (Sigma-Aldrich, St Louis, MO, USA) for 24 h before cell harvesting. Control cells were treated with DMSO that was used to dissolve retinoic acid. Cells were then washed and harvested for subsequent biochemical analysis.

### 4.7. Western Blot Analysis

Two µg of total protein from sample homogenates were separated by SDS-PAGE gels and transferred to a PVDF membrane (Bio-Rad, Hercules, CA, USA) for 1 h in 25 mM Tris-HCl (pH = 8.3), 192 mM Glycine and 20% methanol. Membranes were probed with primary antibody against ALDH enzymes listed in the E. section. Pax-6 (Millipore Sigma—Life Science, Merck KGaA, Darmstadt, Germany) Vimentin (Santa Cruz Biotechnology, Dallas, TX, USA) and Beta Actin (Millipore Sigma—Life Science, Merck KGaA, Darmstadt, Germany). HRP-conjugated secondary antibody was used. Membranes were subjected to Chemiluminescence detection using Luminal according to the manufacturer’s instructions (Amersham Pharmacia Biotech, Piscataway, NJ, USA).

### 4.8. Statistics

Data were obtained from at least three different biological samples. Bars represent mean values ± SEM. *p* values were determined by one-way ANOVA and Student–Newman Keuls for multiple group comparisons (Sigma-Stat softdish, San Francisco, CA, USA). Statistical significance was set at *p* = or < to 0.05. Bar graphs were shown as Mean ± SEM, *n* = 3–5.

## Data Availability

Not applicable.
